# Identification through a transcriptomic approach of candidate genes involved in the adaptation of the cyst nematode *Globodera pallida* to the potato resistance factor *GpaV*_*vrn*_

**DOI:** 10.1186/s12864-025-11332-3

**Published:** 2025-02-24

**Authors:** Océane Lechevalier, Kévin Gazengel, Magali Esquibet, Sylvain Fournet, Eric Grenier, Stéphanie Daval, Josselin Montarry

**Affiliations:** IGEPP, INRAE, Institut Agro, Univ Rennes, Le Rheu, France

**Keywords:** *Globodera pallida*, Virulence, Experimental evolution, Selection, Adaptation, RNAseq, Effectors

## Abstract

**Background:**

Since the banning of chemical products used to control plant-parasitic nematode populations, the use of resistant plants has become the most effective management approach against the potato cyst nematode *Globodera pallida*. However, some populations, from experimental evolution setups and field samplings, are able to overcome these resistances. Herein, a transcriptomics approach was used to disentangle the mechanisms by which *G. pallida* adapts to the plant resistant factor *GpaV*_*vrn*_, and to elucidate the functions involved in this adaptation.

**Results:**

Differential gene expression analysis between virulent and avirulent lineages originating from experimental evolution experiments identified candidate genes involved in the adaptation to *GpaV*_*vrn*_. GO enrichment analyses showed that virulent lineages up-regulated genes involved in cell wall destruction and stress response compared to avirulent lineages. In virulent lineages, a set of genes was up-regulated later in the parasitism stages and are thus potentially involved in adaptation. These genes encode effectors of the VAP and SPRYSEC families contributing to the suppression of plant immunity.

**Conclusion:**

These results will have a major impact on our understanding of the mechanisms by which nematodes adapt to resistant plants, and will contribute to identify effective and sustainable management strategies.

**Supplementary Information:**

The online version contains supplementary material available at 10.1186/s12864-025-11332-3.

## Background

The application of pesticides in general and specifically of nematicides has negative side effects on both the human health and the environment and is therefore not considered as a sustainable solution against pests [1]. Chemical nematicides (e.g. 1,3-Dichloropropene) have been largely used to disinfect soils against nematodes, but were progressively prohibited since 2009 in Europe and have deprived entire agricultural sectors of commercial pest controls. However, the quarantine status attributed to certain nematode species imposes a strict control of contaminated fields, enforcing the use of alternative control strategies. Given nematodes potential for rapid spreading and the consequences for crop yield, the use of resistant plant varieties is of particular interest [2]. Resistant crops offer a specific control method against nematode infestations, reducing dependence on chemical treatments and minimizing the associated environmental risks. Resistant plants have the genetic potential to detect and resist pathogen infection [3]. However, the large-scale and/or the repeated deployment of resistant plants imposes a strong selection pressure leading to a risk of evolution toward virulent populations that can overcome the resistance [4]. The risk of a rapid overcome of plant resistances highlights the need to understand the adaptive mechanisms of nematodes. Understanding nematode adaptive mechanisms is essential for the development of integrated, sustainable and effective control strategies which is crucial for maintaining agricultural productivity.

Among the nematodes threatening the agricultural production worldwide, the cyst nematode *Globodera pallida* is a preoccupying example threatening potato production in many parts of the world. Plant-parasitic nematodes are soil-born crop pest organisms that feed on root tissues. Plant-parasitic nematodes are present worldwide and in all soil types and are responsible for significant economic losses. It is estimated that plant-parasitic nematodes cause yield losses averaging 12.3% worldwide [5, 6]. Moreover, their microscopic size and soil-born nature makes it difficult to detect their presence, and consequently populations are already well established at the time of diagnosis. *Globodera pallida* is a diploid sexually reproducing species which produces one generation per year under European climatic conditions. The hatching of larvae at the second stage juvenile (J2) from the cysts is induced by the perception of hatching factors (HFs) within the exudates secreted by host roots [7, 8]. J2 larvae penetrate roots using their stylets and each larvae establishes a feeding organ called the syncytium through the injection of effectors inside the plant cells [9, 10]. Subsequently, the males emerge from the roots to mate with females, whose body protrude outside the root. When the eggs reach maturity, the females die and form a protective cyst enclosing the next generation of J2, *i.e.* about two-hundred eggs. Both the cyst and the eggshell constitute the survival form of the nematode, protecting J2, which can survive in the soil for more than ten years in the absence of a host plant [11].

In the case of the management of *G. pallida* populations, the overcome of resistant potato cultivars has already been observed in fields in Germany and in The Netherlands [12–14]. Previous studies also demonstrated that *G. pallida* lineages from experimental evolution approaches can adapt to the resistance factors *GpaV*_*vrn*_ (from *Solanum vernei*) and *H3* (from *Solanum tuberosum* ssp. *andigena*) [15, 16]. This situation is preoccupying because most of the European resistant potato cultivars are dependent on the major resistance QTL *GpaV*_*vrn*_ present in the wild species *S. vernei* and located on the potato chromosome V. The resistance conferred by the QTL *GpaV*_*vrn*_ acts by masculinizing nematode populations: it reduces the quality of the syncytium, resulting in the almost exclusive formation of males [17].

Genes encoding effectors are involved in the interaction with the host plant. Effectors are characterized as a set of secreted molecules that manipulate the host for the benefit of the pathogen [18]. Recent progress in the characterization of effector-mediated colonization mechanisms has led to a better understanding of how nematodes parasitize plants [19]. A wide diversity of gene families encoding effectors has been observed [20]. Among the major gene effector families studied are SPRYSEC [21], CLEs [22], HYPs [23] and VAPs [24]. The functions of effectors can be diverse as they may modify plant cell walls, suppress plant immunity or interact with plant signaling. These effectors were mainly identified by genomics, transcriptomics and proteomics studies. They are essential for a compatible interaction between the nematode and the host plant. However, in an incompatible interaction, the product of an effector gene can also be recognized by the product of a plant resistance gene, in accordance with Jones & Dangl’s zig-zag model [25]. This recognition triggers an immune response in the plant. This is why the study of effectors is crucial, even in incompatible interactions. The identification of these effectors will facilitate the comparison of virulent and avirulent nematode populations, which will contribute to a better understanding of their adaptation to the plant resistance genes.

The reduction in sequencing costs thanks to NGS technologies provides opportunities to study plant pathogens using population genomics [26]. Recent advances in the population genomics of plant-parasitic nematodes, combined with progress in NGS, offer the possibility to identify new genes involved in the adaptation to plant resistance [27]. To study the genetic bases of *G. pallida* adaptation to the resistant QTL *GpaV*_*vrn*_, a first GenomeScan approach was performed by sequencing two virulent and two avirulent lineages from an experimental evolution approach. This first population genomics study identified 31 genomic regions displaying markers of selection and containing genes potentially involved in the adaptation [28].

A complementary approach to identify candidate genes involved in the virulence of *G. pallida* relies on transcriptomics that allows to monitor gene expression without a priori. Transcriptome analysis can be used to filter the candidate genes identified by GenomeScan. This filtering facilitates the search for and the selection of candidate genes involved in the adaptation. Together, these approaches could allow to understand both the genetic basis of virulence and the molecular mechanisms involved.

RNAseq studies have already been performed on the potato cyst nematode *G. pallida*. A transcriptomic analysis was performed to examine changes in gene expression throughout the life cycle of *G. pallida*, providing important information, specifically on genes involved in root invasion and feeding site establishment. Large changes in gene expression between the different life stages were observed, with a higher number of genes expressed on mobile J2 and male stages while a decrease was observed during the later stages of development [29]. Transcriptomes of all stages from dry cysts to hatched juveniles were also compared using RNAseq showing that genes linked to an increase of calcium and water uptake were up-regulated during transition from survival to hatching [30]. Another study suggested that host specificity is determined by the regulation of essential effectors and may be under the control of a single or very few regulatory genes [31]. Transcriptomes of *G. pallida* were also compared between populations from two distinct host species, the susceptible *Solanum tuberosum* and the resistant *Solanum sisymbriifolium*, and displayed contrasts in the expression of transcripts involved in plant parasitism [32].

The present transcriptomic study aims to use *G. pallida* lineages obtained from experimental evolution to identify candidate genes involved in the nematode adaptation to potato resistances. The nematode lineages were phenotyped and their transcriptomes were analyzed to identify genes differentially expressed between virulent and avirulent lineages. This comprehensive approach validates the lineages’ virulence status and the changes in gene expression between avirulent and virulent nematode lineages through the identification of genes involved in plant parasitism and encoded as effectors.

## Results

### Virulence level of the different *G. pallida* lineages

**Fig. 1:**
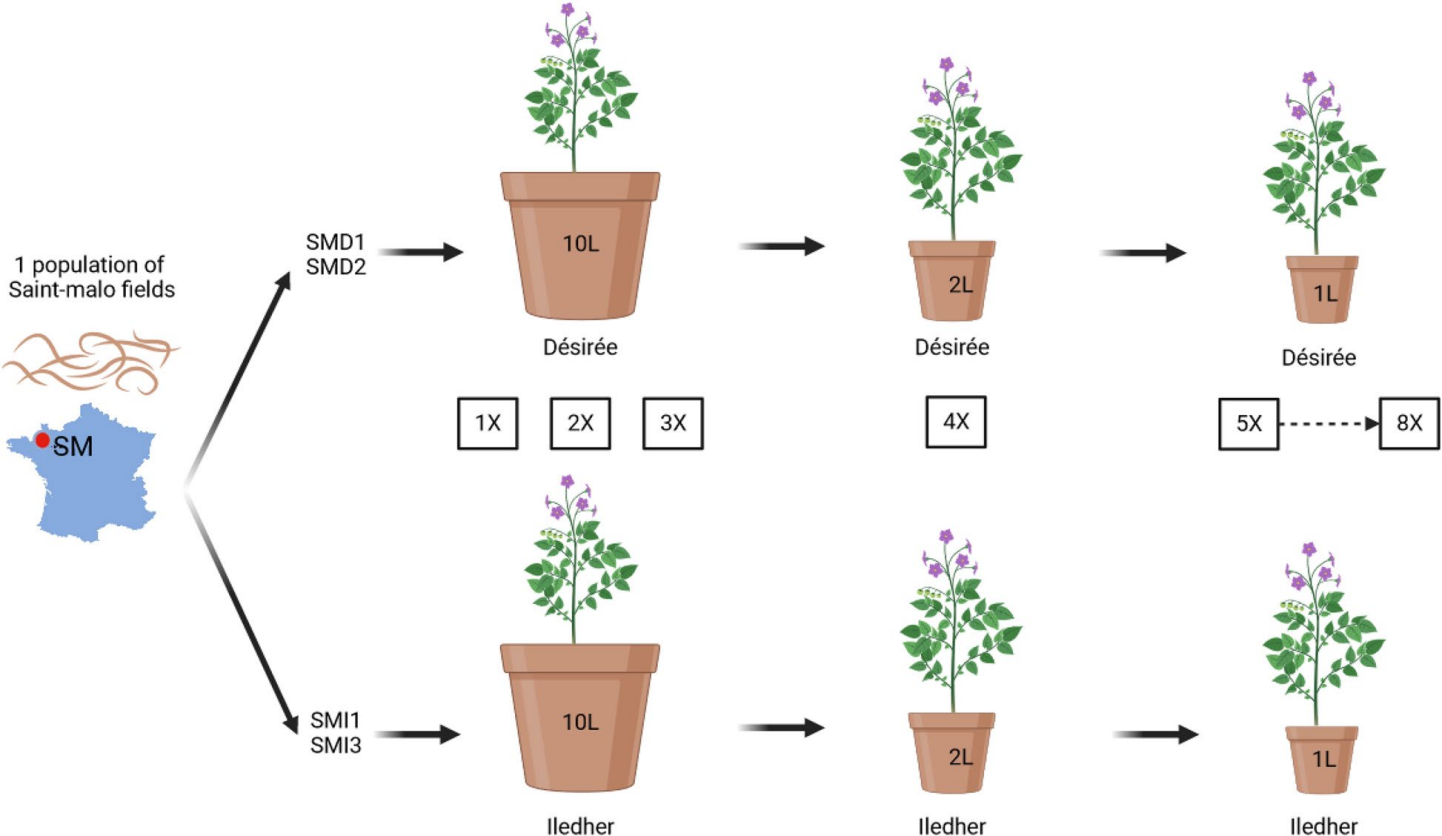
Experimental design. The four lineages were obtained from a natural population and evolved during eight generations on the susceptible potato cultivar Désirée (SMD1, SMD2) or on the resistant potato cultivar Iledher (SMI1, SMI3)

The four *G. pallida* lineages used in this study were obtained through an experimental evolution approach performed in greenhouse from a natural population. Two lineages (SMD1 and SMD2) were independently confronted to the susceptible potato cultivar Désirée during eight generations (*i.e.* eight years as *G. pallida* performed one generation per year under European conditions), while the two others (SMI1 and SMI3) were confronted to the resistant cultivar Iledher which harboured the resistant QTL *GpaV*_*vrn*_ from *S. vernei* (Fig. 1). The virulence level of each lineage was measured on both susceptible and resistant potato cultivars (Désirée and Iledher, respectively). To do so, juveniles were inoculated on potato roots in petri dishes and the percentage of females produced was monitored.

**Fig. 2:**
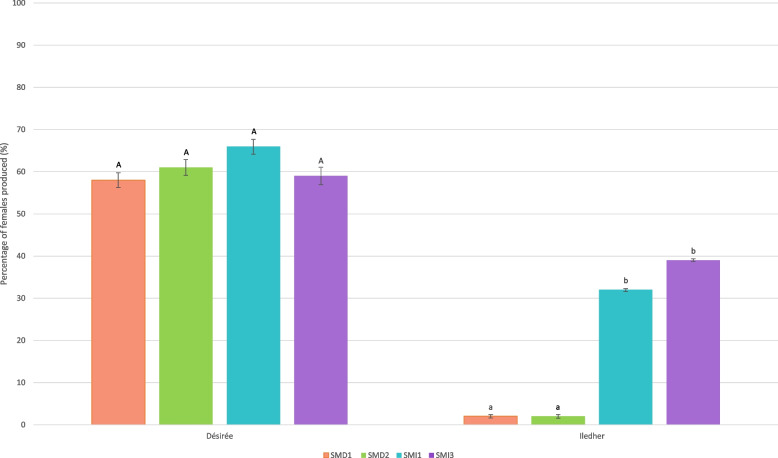
Percentage of females produced on the susceptible potato cultivar Désirée and the resistant potato cultivar Iledher for each *Globodera pallida* lineage after eight generations on the susceptible (SMD1, SMD2) or on the resistant (SMI1, SMI3) potato cultivar. Letters represent the homogenous groups identified by the Tukey test at the 5% threshold

The average percentage of females obtained for the four lineages on the susceptible cultivar Désirée reached 60% and there was no significant lineage effect (F_3,83_ = 0.78 and *P* = 0.51; Fig. 2). This result confirmed that all studied lineages were able to produce females on the susceptible potato cultivar.

On the resistant cultivar Iledher, a significant difference in female production was observed between SMD and SMI lineages (F_3,72_ = 29.20 and *P* < 0.0001; Fig. 2). An average of 2% females were produced by the SMD1 and SMD2 lineages on the resistant potato Iledher, highlighting the high efficiency of this masculinizing resistance. However, the production of females by the lineages SMI1 and SMI3 reached on average 35%: this result clearly showed that after eight generations on the resistant cultivar, those lineages were partially adapted (virulent) to the resistance conferred by the major QTL *GpaV*_*vrn*_.

These phenotyping results enabled to determine the virulence status of the lineages in order to study the mechanisms involved in the adaptation of *G. pallida* to the potato resistance factor *GpaV*_*vrn*_.

### Overview, mapping and validation of RNAseq data

Eight RNA samples, *i.e.* four lineages with two replicates per lineage (A and B), were sequenced on one SP lane of the Illumina Novaseq technology. Quality control of the sequences revealed good quality scores for all sequences, with an average PHRED score of 30 over the entire reads’ length in all samples. An average of 48 million paired-end reads (2 × 150 bp) per sample was obtained and mapped to the reference genome G_Pallida_D383_v.0.8.1 [33].

A total of 18,071 genes were obtained at the start of the analyses. This number was reduced to 11,680 genes according to the CPM threshold applied, to select genes with enough read to compute a differential expression test. The PCA displays two groups of samples, SMI3B, SMI1A and SMI1B on one hand and SMD2B, SMD1A and SMD1B on the other hand, and two outlier samples, SMI3A and SMD2A (Fig. S1A). These two outlier samples were discarded from the dataset before further analyses. Without these two samples, virulent and avirulent lineages were clearly separated on the PCA (Fig. S1B and Fig. S2).

### Virulence-related differential gene expression

**Fig. 3:**
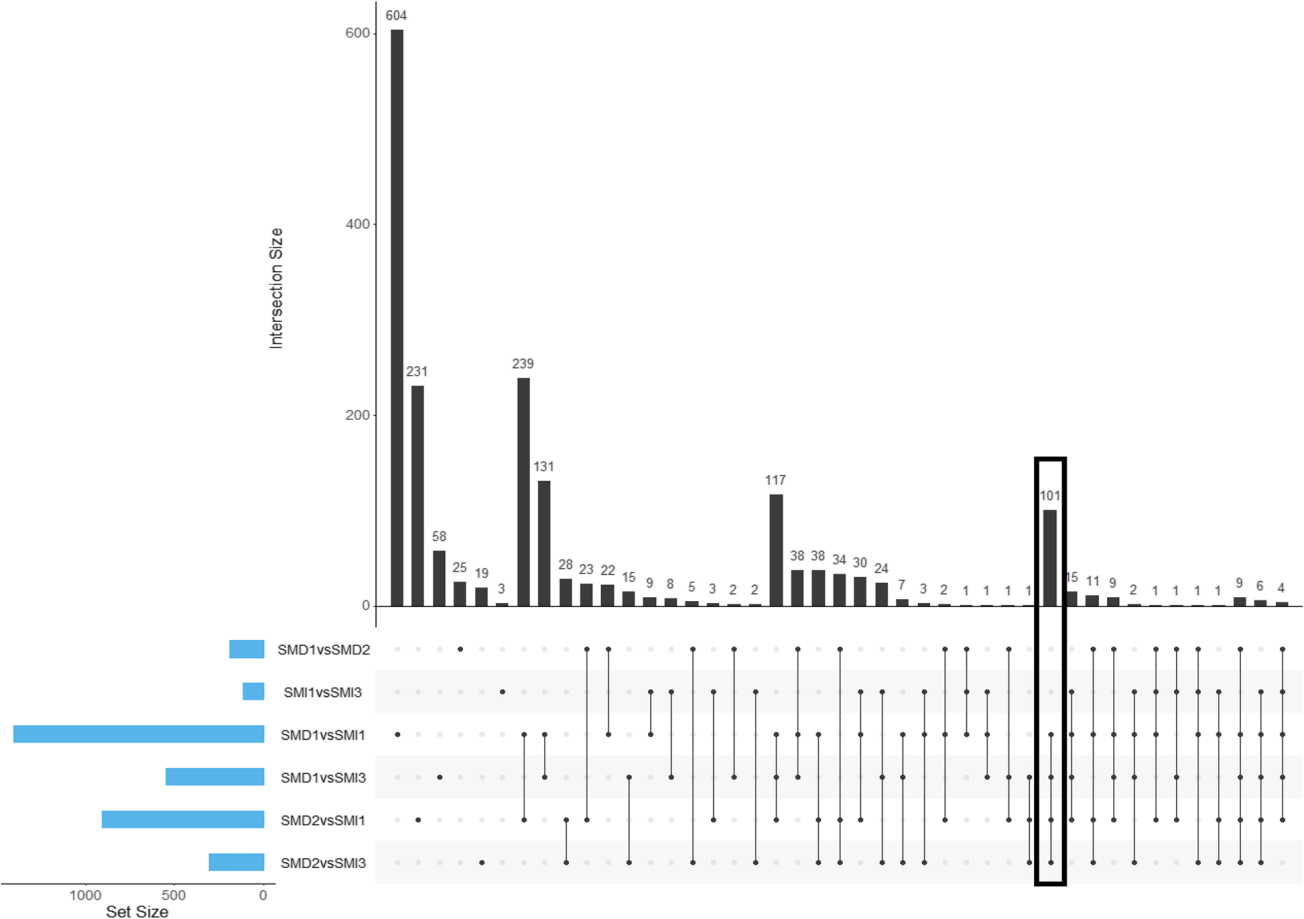
Plot of intersections between sets of genes differentially expressed. The total number of Differentially Expressed Genes (DEGs) for each comparison is presented in the horizontal bars on the left. Dots show the presence of DEGs in each comparison and the lines connecting dots represent the intersections of gene lists between comparisons. The vertical bars and associated numbers correspond to overlap of DEG sets

Expression analyses identified 1291 Differentially Expressed Genes (DEGs) according to the different lineages. Among them, 674 genes were up-regulated and 617 were down-regulated in virulent lineages (SMI) compared to avirulent lineages (SMD). Genes were selected by focusing on the DEGs found in the four following comparisons: SMD1 versus SMI1 or SMI3 and SMD2 versus SMI1 or SMI3. DEG comparisons between SMD1 and SMD2 and between SMI1 and SMI3 were not considered, as the study was only interested in the comparison between virulence and avirulence conditions. The 101 genes satisfying these conditions are shown in Fig. 3 and described in Table S1.

### Functional characterization of differentially expressed genes

**Fig. 4:**
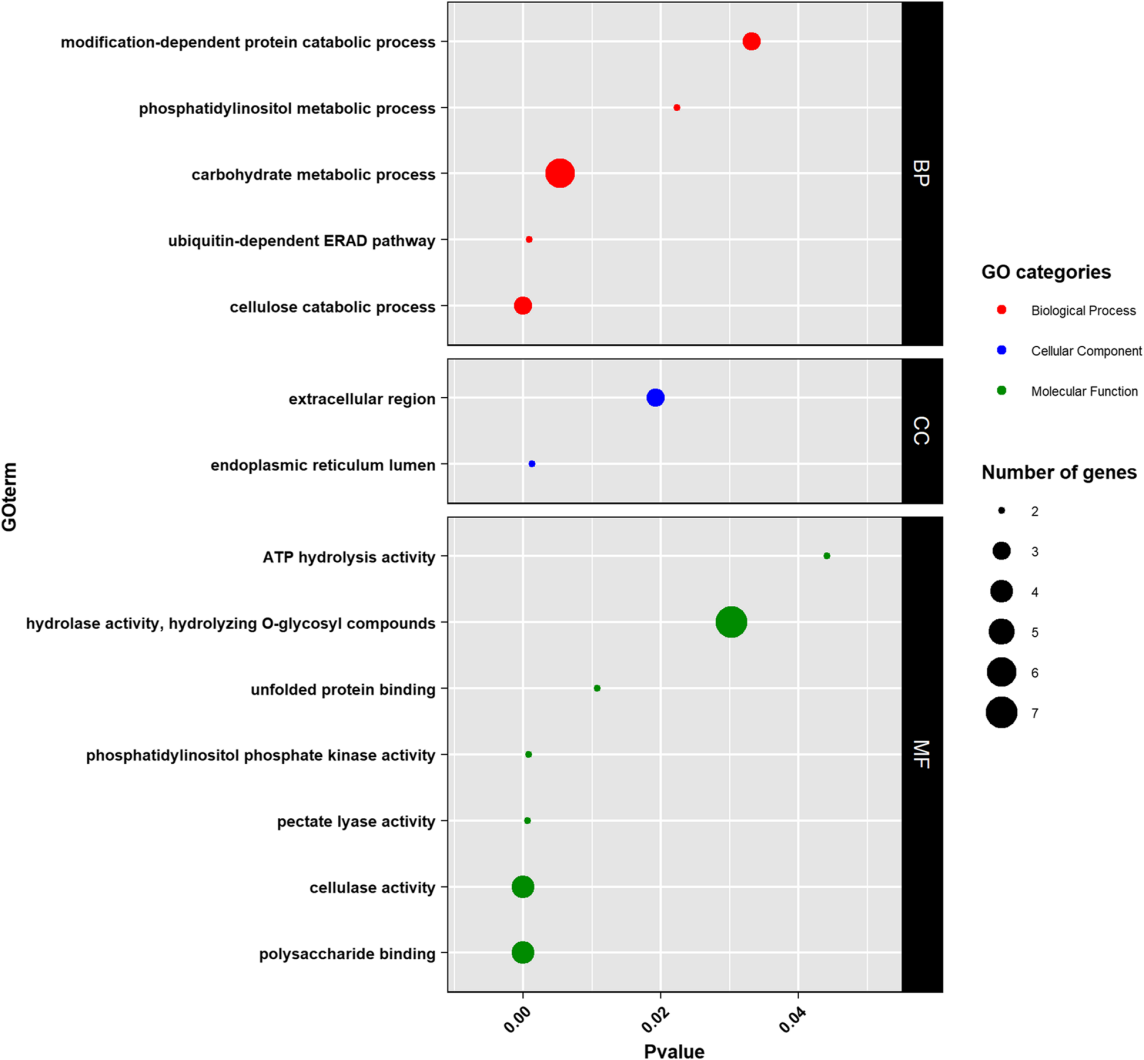
GO-term enrichment on the 101 DEGs between *Globodera pallida* lineages. Y-axis represents enriched GO-terms for each ontology category (BP = Biological Process, CC = Cellular Component and MF = Molecular Function). X-axis shows *p*-values for each enriched GO-term. A larger circle diameter highlights higher number of genes that have enriched the term

To better understand how the transcriptome differed between the lineages, a Gene Ontology (GO) terms enrichment analysis was achieved on the list of 101 DEGs. In total, 5 biological processes, 2 cellular components and 7 molecular functions were significantly enriched (Fig. 4). The major terms (*i.e.* based on both *p*-value and number of genes explaining the enrichment) included functions linked to cellulase activities or carbohydrate catabolic activities.

The 101 DEGs selected are all associated with GO terms. To go further, enriched GO-terms were also analyzed within the lists of up-regulated and down-regulated genes in SMI compared to SMD (Fig. S3).

### Clustering of differentially expressed genes

**Fig. 5:**
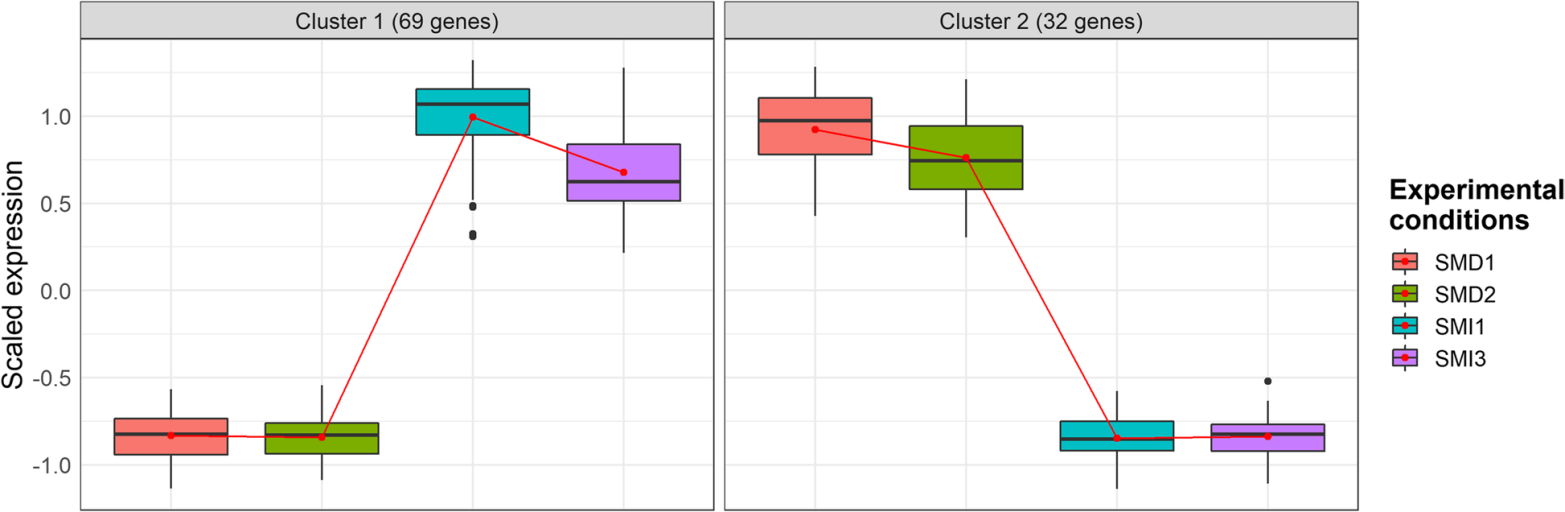
Clustering of DEGs. Cluster 1 represents the up-regulated genes and cluster 2 the down-regulated genes in SMI (virulent) compared to SMD (avirulent). X-axis shows each experimental condition using colored boxes. Y-axis represents scaled gene expression

These 101 genes were then clustered, using coseq package through AskoR tool, to group genes with similar expression profiles (Fig. 5). Two distinct clusters were observed, separating SMD and SMI lineages. The cluster 1 included 69 up-regulated genes in SMI (virulent) compared to SMD (avirulent) While the cluster 2 included 32 genes down-regulated in SMI (virulent) compared to SMD (avirulent).

### Selection of candidate genes

The presence of a signal peptide, revealing the potential secretion of effectors by J2 larvae, was tested using SignalP on each of lists containing i) the 11,680 starting genes, ii) the 1292 DEGs, and iii) the selection of 101 genes of interest. Interestingly, 13.6% of the 11,680 starting genes contained a peptide signal as well as for the 1292 DEGs. In contrast, the percentage of genes containing a signal peptide was 49.5% on the selection of 101 genes (*i.e.* 50 genes) indicating that this selection was enriched in genes with a peptide signal compared to the overall genome.

We thus selected as candidates the DEGs that contained a signal peptide for a total of 50 genes. From this selection, 25 genes were discarded because they encoded unknown proteins. A supplementary Blast search (PSI-BLAST, BLASTN, BLASTP) was not able to find a function for these proteins. Among the 25 candidates, 21 were up-regulated and 4 down-regulated in SMI virulent lineages compared to SMD avirulent lineages, respectively. In this selection, 7 genes were involved in cell degradation, 3 were linked to nutrient uptake by the nematode, 2 to the response of stress caused by the plant’s immune response, and 8 were annotated with other functions. Importantly, 5 genes were involved in the suppression of plant immunity (Table 1). The 4 genes down-regulated were all annotated as coding for proteins with others functions. The functions of up-regulated genes are particularly interesting in relation with the nematode adaptation to plant resistance. Recognition of the effectors by the plant would require the nematode to compensate by producing more of these effectors in order to effectively suppress the immunity of the resistant plant. The 5 candidate genes selected were previously characterized as nematode effectors which manipulate the host: *VAP1* [24], and 4 SPRYSEC (18, 19, 5 and *RBP5*) [21, 34, 35]. The genes *VAP1* and *SPRYSEC-19* were also characterized as virulence genes whose product is capable of interacting with a tomato resistance gene [24, 36]. The gene *RPB5* also named *Gp-SPRY33H17* can suppress, under certain conditions, the characteristic cell death induced by the recognition of a secreted nematode protein RBP-1 by the potato resistance gene *Gpa2* [37].

**Table 1:** Candidate genes potentially involved in suppressing plant immunity. Those five genes are up-regulated in the virulent lineage (SMI). The correspondence with Gene ID from the reference genome from Cotton [29] is indicated

Gene ID D383	Protein Name	Fold Change	Reference	Gene ID Lindley
Gpal_D383_g07991	VAP1 protein	1.68	Lozano-Torres, J.L. et al., 2014 [24]	GPLIN_001139400
Gpal_D383_g12448	RBP5 protein	2.82	Diaz-Granados, A. et al., 2016 [21]	GPLIN_000657200
Gpal_D383_g15313	Secreted SPRY domain containing protein 18	2.34	Diaz-Granados, A. et al., 2016 [21]	GPLIN_000507800
Gpal_D383_g15316	Secreted SPRY domaincontaining protein 19	2.58	Postma, W.J. et al., 2012 [34]	GPLIN_000892900
Gpal_D383_g16477	Secreted SPRY domaincontaining protein 5	2.42	Diaz-Granados, A. et al., 2016 [21]	GPLIN_001206200

## Discussion

In this study, we aimed to identify genes responsible for the adaptation of the cyst nematode *G. pallida* to potato resistance, by combining experimental evolution and transcriptomic approaches. After phenotypically validating the adaptation of lineages to the resistant variety, differential gene expression analyses between the transcriptomes of virulent and avirulent lineages enabled the identification of candidate genes.

The four independent lineages obtained from experimental evolution were phenotyped on both potato cultivars. On the susceptible plant cultivar Désirée, all nematode lineages showed a female production of around 60%. On susceptible cultivar Iledher, both nematode lineages multiplied for eight generations (SMD1 and SMD2), were avirulent and produced very few females, as expected. On the other hand, the lineages that have been confronted to the resistant cultivar (SMI1 and SMI3) were able to produce females on this same resistant cultivar, demonstrating their ability to adapt to the resistance. However, the production of females (35%) remained lower on the resistant cultivar than on the susceptible one, demonstrating a partial adaptation after eight generations. The virulence allele(s) was(were) probably not yet fully fixed in these lineages.

The transcriptomes of the hatched J2 larvae of two avirulent and two virulent lineages were profiled, with two replicates per lineage. Using RNAseq sequencing data from eight samples, the distance between transcriptomes was analyzed by PCA. As expected, a strong separation on the X-axis was observed according to lineage virulence. However, two samples (SMD2A, SMI3A) were distant from the others on the Y-axis. As no biological explanation was identified, these two samples were removed before further analyses. This filtering nevertheless allowed to keep three avirulent and three virulent samples. All the analyses were performed using for both the 6-samples dataset and the 8-samples dataset and the results provided similar lists of candidate genes (data not shown).

With the 6-samples dataset, 101 DEGs were detected between virulent and avirulent lineages. These 101 genes being common to all independent comparisons, this assumed that the same genes were involved in the resistance adaptation of the two independent evolutionary lineages. In order to identify their potential functions, a GO-term enrichment was performed. The enrichment analysis highlighted terms related to nematode ability to develop on the resistant cultivar: terms involved in cellular degradation, interaction with cellulose and glucose.

The list of 101 DEGs (Table S1) was refined by focusing on genes for which a signal peptide was detected. The presence of this short peptide in the N-terminus of neosynthesized proteins reveals their link with the secretory pathway. The presence of this peptide chain, marker of secreted proteins, suggests an interaction between the nematode and the plant [10]. The proportion of genes showing signal peptides in the 101 DEGs was significantly higher than in the rest of the genome. This suggests that they are good candidate genes that could be involved in the infection process. As a result, 50 of the 101 genes were selected for further investigation.

Among the 50 genes, 25 had no functional annotation, even using various databases (PSI-BLAST, BLASTN, BLASTP, PPND). This could be improved by further analyses like 3D protein structures of the 25 unannotated genes using Alphafold2 [38] in order to compare the structure with that of proteins from *Caenorhabditis elegans*, a nematode largely studied with complete genome annotations.

The remaining 25 annotated genes were studied individually and compared with the existing literature. Among these genes, many were characterized as cellulases and effectors in previous studies [19]. The effectors secreted by the parasitic organism influence the plant immune response in favor of parasitism [39].

The proteins required for root invasion are secreted in cells of the subventral esophageal gland. It has been shown in plant-parasitic nematodes that effectors produced in the subventral salivary gland (1,4-endoglucanases, pectate lyases, expansins) are secreted earlier in the interaction than those produced in the dorsal salivary gland [40]. These are therefore candidates for effectors involved in the nematode’s ability to penetrate and migrate into the potato root. This is the case for the gene coding for the GR-EXPB1 protein already identified in the potato cyst nematode *Globodera rostochiensis*. This nematode has been shown to produce a functional expansin generally found in plants. This GR-EXPB1 expansin is used to disrupt cell walls during invasion of the host plant [41]. Genes encoding proteins able to degrade plant cell walls were also found, such as the 1,4-endoglucanase [42], the pectate lyase [43] or other cellulases. 1,4 endoglucanases, the first effectors identified from PPN, have been identified in *G. rostochiensis* and are produced in the cells of the subventral esophageal gland. These effectors degrade β-1,4-glucan polysaccharides such as cellulose, and are therefore involved in the hydrolysis of plant walls, likely facilitating intracellular migration of the nematode through plant roots [44]. Pectate lyase 1 has previously been studied in the root-knot nematode *Meloidogyne graminicola* and was exclusively expressed in cells of the subventral esophageal gland. It degrades cell walls and increases the pathogenicity of nematodes [45]. These different proteins were consistent with the observed enriched GO-terms involved in cell wall degradation. They were all up-regulated in SMI virulent lineages with a fold-change greater than 1.56.

However, the above-mentioned genes act early in the colonization process and are thus not necessarily the best candidates for resistance adaptation. Because this resistance acts during the establishment and/or growth of the syncytium, which causes masculinization, the plant’s recognition of the avirulence factor is supposed to occur at later stages of infection. The cells of the dorsal esophageal gland are more specialized in secretion during sedentary stages, most likely producing effectors involved in the formation and maintenance of the feeding site. We therefore need to focus on gene products secreted by the dorsal salivary gland, which are secreted later and can therefore interact with the suppression of the plant immune response [18].

The secretion of effectors within plant tissues induces plant immune responses. It is therefore possible that these plant responses induce stress in the nematode and justify the presence of proteins such as Heat Shock Protein (HSP) 70 or 90 with a Fold Change of 1.79 and 1.58, respectively. This is a chaperone protein that ensures the correct conformation cell’s proteins, protecting cells from extreme stress.

Five candidate genes potentially involved in the adaptation of *G. pallida* to potato resistance conferred by the *GpaV*_*vrn*_ QTL have been identified. They are known to be involved in the suppression of the innate plant immune system. The gene encoding a venom allergen-like protein (VAP1) has already been studied in *G. rostochiensis*. This allergenic protein has been shown to selectively suppress host immunity mediated by immune receptors located on the plant surface [24]. Nematodes therefore most likely use allergen-like proteins to suppress the activation of defenses by immunogenic degradation products in damaged host tissue. Furthermore, phylogenetic trees reveal that the VAP protein we identified in *G. pallida* does indeed have an ortholog in *G. rostochiensis* [33].

Another important effector family for resistance adaptation is the SPRYSEC family. Expression of SPRYSEC genes in potato cyst nematodes is specifically localized in the dorsal esophageal gland cell and disrupts the plant’s defenses [37, 46, 47]. Twenty-four and 60 paralogs belonging to this family have been described in *G. pallida* and *G. rostochiensis*, respectively [33]. Four genes encoding this family have been identified in the present study: *SPRYSEC-5*, *SPRYSEC-18*, *SPRYSEC-19* and *RBP5*, which is also a member of the SPRYSEC family. They all had a fold-change greater than 2.31 and were up-regulated in SMI virulent lineages. These four effectors belong to the two phylogenetic groups described in the SPRYSEC family [33] with *SPRYSEC-18*, *SPRYSEC-19* belonging to clade A and *SPRYSEC-5* and *RBP-5* belonging to clade B which also harbors the well-known *RBP-1* virulence gene. SPRYSEC are known to be involved in both suppression and activation of resistance genes-mediated plant immunity. The polyvalence of the SPRY domain as a protein-binding module enables nematodes to disrupt various host protein complexes required for plant immunity [21]. *SPRYSEC-19* of *G. rostochiensis* has been shown to function as a suppressor of programmed cell death and disease resistance [34]. *RBP-5* of *G. pallida* is the closest paralog to *RBP-1* (Gpal_D383_g12854) identified as the protein triggering immunity in the presence of the *Gpa2* potato resistance gene [46]. *RBP-5* was also described as an effector with a nuclear subcellular localization, suggesting that it may be important in modifying plant gene expression or in regulating changes in the cell cycle during the syncytium formation [35]. These genes are therefore good candidates for the adaptation of nematode populations to plant resistance.

It is therefore consistent to find, as candidates, effectors identified as able to suppress either plant basal immunity (*VAP1*) but more importantly effector triggered immunity (SPRYSEC). This suggest that the genes identified herein are likely not recognized by the *GpaV*_*vrn*_ resistance but that the adaptation process is linked to the ability to overexpress particular suppressors preventing plant cell death allowing the development of a syncytium able to provide enough nutrients to the larvae for their development towards a female adult stage. The candidate genes obtained, encoding the above-mentioned effectors, explain part of the nematode’s ability to circumvent the plant’s resistance. A complex interaction takes place between the plant effector triggered immunity and the effectors acting as suppressors secreted via the nematode stylet. The mechanisms of interaction with the plant are probably caused by a combination of several nematode effectors, which can differ according to the stage of parasitism and the plant response. Once the first line of physical defenses has been crossed, such as cell walls, the nematode faces a second line based on recognition of the pathogen.

To date, few genes have been identified as clearly involved in nematode adaptation to plant resistance. The five candidate genes obtained here using a transcriptomic approach are potentially involved in this adaptation process, and provide insight into the mechanisms of interactions between nematodes and plant resistance gene products. To compare these candidate genes with nematode genomic regions highlighted in previous studies, we have identified for the orthologues of our five candidate genes on the reference genome produced by Cotton et al. [29]. The five GPLIN genes corresponding to the five candidates of this study were compared with the lists provided by Eoche-Bosy et al. [28] and Varypatakis et al. [16]. While none of them correspond to the genes identified under the 31 candidate regions of Eoche-Bosy et al. [28], one SPRYSEC gene (Gpal_D383_g15313 which corresponds to GPLIN_000507800 in the reference genome produced by Cotton et al. [29]) was also identified as a good candidate in the list of Varypatakis et al. [16]. This gene was thus identified as a candidate Avr gene associated with *H3* resistance [16] and with *GpaV*_*vrn*_ resistance (the present work), suggesting that this gene could be important in the interaction with resistant plants but not directly involved in the recognition process. Indeed, it is unlikely that the selection for virulence to distinct resistant genes acts on the same effector. Anyway, whatever the used approach, *i.e.* transcriptomic, whole-genome scanning [16, 28] or pathogen enrichment sequencing—PenSeq [16], the three lists of candidate Avr genes are clearly enriched in SPRYSEC genes.

## Conclusions

The identification of genes and genomic regions associated with nematode adaptation to potato resistances is a promising new perspective for a better understanding of the mechanisms behind adaptation. This understanding will enable the development of specific diagnostic tools to detect and monitor the presence of virulent nematodes in crops. Anticipating the overcoming of resistance is essential to identify optimal sustainable management strategies. By integrating these strategies into farming practices, farmers will be able to reduce their dependence on pesticides, thus contributing to the preservation of soil health and the overall sustainability of cropping systems.

## Methods

### Experimental design

The nematode lineages used in this study were obtained through an experimental evolutionary approach conducted over eight years (Fig. 1). As *G. pallida* only produces one generation per year, experimental evolution resulted in eight generations. The lineages were obtained from a natural population from a field near Saint-Malo (SM) in France which was repetitively confronted to two potato cultivars. The first one, Désirée, is a susceptible cultivar, and the second one, Iledher, is a resistant cultivar. The latter harbours the *GpaV*_*vrn*_ QTL, a resistant major QTL derived from the *S. vernei* resistance source, which act by masculinizing nematode populations [17]. This experiment was conducted in greenhouse with independent replicates: SMD1 and SMD2 evolved on Désirée, SMI1 and SMI3 evolved on Iledher. The first three generations were produced in 10L pots with three tubers per pot. All cysts from generation 3 were extracted using a Kort elutriator and pooled together in a single tulle bag, allowing juveniles going through but retaining cysts, for each replicate and placed in 2L pots to perform the generation 4. From generation 5 to generation 8, 1L pots were used and cysts were extracted each year. The number of inoculated cysts per pot was variable, from 143 to 1000, depending on the number of cysts available from the previous generation. For the last generation, the number of inoculated cysts was standardized at 200 cysts.

### Phenotyping

Virulence levels were measured in Petri dishes following the protocol described by Fournet et al. [15]. Stimulation of J2 larvae hatching was induced by placing 10 cysts per lineage in potato root exudates (cv. Désirée) during eight days. Pieces of potato tuber were deposited on agar, allowing young roots to develop for 3 days. Ten newly hatched J2 were inoculated per root apex, and 20 roots were used per condition, for a total of 200 larvae per condition. The four lineages were inoculated onto both potato cultivars, Désirée and Iledher, representing eight conditions. After 18 days, the roots were dissected under a binocular magnifying glass to count males and females for each condition (Fig. S4). All steps (hatching, root formation and nematode development) were carried out in the dark at a temperature between 17 °C and 22 °C, for all lineages and for both potato cultivars, which were therefore exposed to the same environmental conditions.

Statistical analysis of phenotyping data was carried out using R software (v4.2.2). After checking for equality of variance and normality of residuals (Levene and Shapiro–Wilk tests, respectively), a one-way ANOVA was carried out on the number of females produced to test the lineage effect independently on each potato cultivar. In the event of a significant effect, a multiple comparison of means test was performed (Tukey test).

### RNA extraction and sequencing

RNA extraction was performed on hatched J2 larvae from 300 cysts. Hatching was stimulated with potato root exudates (cv. Désirée). Every two days, the hatched larvae were collected, the cysts cleaned and the exudates changed to limit contaminating bacterial development. Larvae are then frozen before RNA extraction according to the protocol of Sabeh et al. [31] using the Qiagen Rneasy mini-kit. Lineages were separated into two batches and separate extractions were performed on each batch to have two replicates per lineage (A and B), for a total of eight samples from 300 cysts for each. RNA quality and quantity were estimated using Agilent 2100 Bioanalyzer System technology. The average amount of total RNA obtained was 2.7 μg per sample. The eight messenger RNA libraries were sequenced on a 2 × 150 bp SP line of Illumina’s Novaseq high-throughput sequencer by the GeT-PlaGe genomics platform (Toulouse, France). The libraries were obtained with TruSeq Stranded RNA.

### Read processing, mapping, and counting

Sequencing data were analyzed on the GenOuest cluster (Rennes, France). They were subjected to quality control using FastQC v.0.11.7 (Babraham Bioinformatics—FastQC), a quality control tool for high-throughput sequence data. Read quality above Q30 required no further processing.

Then, reads were mapped to the reference genome G_Pallida_D383_v.0.8.1. This reference genome of 113 Mb contains 163 scaffolds [33]. Reads were mapped using the STAR tool version 2.7.2b12 [48], with default parameters. FeatureCounts v1.6.013 [49] tool was used with the GFF annotation file to count the number of reads on each *G. pallida* gene mapped to the reference genome.

### Identification of differentially expressed genes

AskorR tool (https://github.com/asusete/askoR/) was used with R program 4.2.2 to perform differential gene expression analysis. This tool is implemented with edgeR package [50] to determine Differentially Expressed Genes (DEGs), coseq package [51] for gene clustering, and upsetR [52] and ggplot2 packages (https://ggplot2.tidyverse.org) for representation of gene lists intersections. Genes that were counted less than one count per million (CPM) in less than 3 samples were eliminated for bias reduction. Data was normalized using the default TMM (Trimmed Mean of M values) method of edgeR. To visualize and analyze reproducibility between replicates, a Principal Component Analysis (PCA) was performed, showing dispersion between samples based on dimension reduction. A heatmap was used to visualise the samples selected according to CPM.

DEGs were selected according to a FDR < 0.05. No logFC (Fold-Change) threshold was applied in order to select all DEGs, including those with moderate expression ratios.

### Gene Ontology term (GO-term) enrichment

Functional characterization of transcripts was performed using Blast2go tool 1.5.1 [53] on GenOuest cluster to identify GO-terms affiliated to each gene. The topGO package implemented in AskoR was used for GO-term enrichment. Enrichment analyses were performed with weigth01 algorithm, Fisher’s exact test, and nodeSize set to 10 (to remove GO-terms represented with less than 10 genes in the genome). A *p*-value < 0.05 was applied to define significantly enriched GO terms. For each GO category (Molecular Function, Cellular Component, and Biological Process), top enriched GO-terms with fold enrichment ratios that were enriched with at least 2 genes were represented in graphs.

### Gene clustering

AskoR also integrates gene clustering tool coseq package [51] for group genes that have similar expression profiles. The mode kmeans and clr transformation were applied to clustering genes into groups.

### Selection of candidate effectors

Candidate effectors were identified from the DEGs and their annotation. The effector characteristic has been predicted by the presence of signal peptides, specifics of secreted peptides, using SignalP v6.0 [54]. To identify the correct homolog of the best candidate effector genes from the *G. pallida* assembly produced by Cotton et al. [29], we performed a homolog search using the WormBase ParaSite website [55] and the Miniprot software [56].

## Supplementary Information

Supplementary Material 1.

## Data Availability

Raw reads of the 8 samples are available at the European Nucleotide Archive database system under the project accession number PRJEB76451 (https://www.ebi.ac.uk/ena/browser/view/PRJEB76451).
