# Supplementary material for: Identification through a transcriptomic approach of candidate genes involved in the adaptation of the cyst nematode Globodera pallida to the potato resistance factor GpaVvrn
Source: BMC Genomics. 2025 Feb 24;26:191. doi: 10.1186/s12864-025-11332-3 (PMC11852814; doi:10.1186/s12864-025-11332-3)

**Identification through a transcriptomic approach of candidate genes** **involved in the adaptation of the cyst nematode *Globodera pallida* to the potato resistance factor *GpaV_vrn_***

Océane LECHEVALIER, Kévin GAZENGEL, Magali ESQUIBET, Sylvain FOURNET, Eric GRENIER, Stéphanie DAVAL^$^ and Josselin MONTARRY^$*^

IGEPP, INRAE, Institut Agro, Univ Rennes, Le Rheu, France

^$^ These authors contributed equally to this work

^*^ Corresponding author: josselin.montarry@inrae.fr

**Supporting Information**

**Table S1** **–** Selection of 101 DEGs obtained from AskoR and potentially involved in adaptation.

| **Gene_ID** | **Prediction** | **Expression** | **FC** | **Annotation** | **Chr** | **Length** |
| --- | --- | --- | --- | --- | --- | --- |
| Gpal_D383_g00017 | SP | SMD<SMI | 1.8 | NA | scaffold1 | 1927 |
| Gpal_D383_g00020 | SP | SMD<SMI | 1.63 | NA | scaffold1 | 407 |
| Gpal_D383_g00185 | NA | SMD<SMI | 1.57 | NA | scaffold1 | 3192 |
| Gpal_D383_g00431 | SP | SMD<SMI | 1.66 | NA | scaffold1 | 623 |
| Gpal_D383_g00436 | NA | SMD<SMI | 1.74 | PIO77251.1phosphoglycerate mutase family protein | scaffold1 | 1749 |
| Gpal_D383_g00537 | SP | SMD>SMI | 1.54 | CDJ96568.1Protein C39E9.8, isoform d | scaffold1 | 1922 |
| Gpal_D383_g01237 | NA | SMD<SMI | 1.43 | NA | scaffold1 | 1196 |
| Gpal_D383_g01268 | NA | SMD>SMI | 2.83 | KAF7637437.1PHB domain-containing protein | scaffold1 | 973 |
| Gpal_D383_g02690 | NA | SMD>SMI | 1.66 | OZC11640.1RNA helicase HRH1 domain protein | scaffold2 | 4911 |
| Gpal_D383_g02789 | NA | SMD>SMI | 2.08 | CAB48391.1 peroxiredoxin | scaffold2 | 797 |
| Gpal_D383_g03360 | NA | SMD>SMI | 1.44 | KHN85462.1Mitochondrial import inner membrane translocase subunit tim-8 | scaffold3 | 1134 |
| Gpal_D383_g03921 | NA | SMD<SMI | 1.68 | KAF7635707.1EF-hand domain-containing protein | scaffold3 | 2089 |
| Gpal_D383_g04056 | SP | SMD<SMI | 1.56 | CAC83611.1EXPB1 protein | scaffold3 | 1097 |
| Gpal_D383_g04325 | SP | SMD<SMI | 2.23 | NA | scaffold3 | 5974 |
| Gpal_D383_g04400 | SP | SMD<SMI | 2.02 | AHW98760.1beta-1,4-endoglucanase precursor | scaffold4 | 1569 |
| Gpal_D383_g04455 | NA | SMD<SMI | 2.05 | KAF7635199.1PIPK domain-containing protein | scaffold4 | 1336 |
| Gpal_D383_g04470 | NA | SMD>SMI | 4.35 | CAD2179409.1unnamed protein product | scaffold4 | 360 |
| Gpal_D383_g04665 | SP | SMD<SMI | 2.57 | WP_191190711.1FAD-binding protein | scaffold4 | 2279 |
| Gpal_D383_g04715 | NA | SMD<SMI | 1.63 | AAC63989.1beta-1,4-endoglucanase precursor | scaffold4 | 3143 |
| Gpal_D383_g04716 | SP | SMD<SMI | 1.85 | AAC63989.1beta-1,4-endoglucanase precursor | scaffold4 | 1481 |
| Gpal_D383_g05253 | NA | SMD<SMI | 1.95 | KAF7640495.1Protein kinase domain-containing protein | scaffold4 | 2123 |
| Gpal_D383_g05790 | SP | SMD<SMI | 1.58 | CAP26348.2Protein CBG06014 | scaffold5 | 3724 |
| Gpal_D383_g06079 | NA | SMD<SMI | 1.63 | KAF7639332.1LIM zinc-binding domain-containing protein | scaffold5 | 940 |
| Gpal_D383_g06263 | SP | SMD<SMI | 1.65 | NA | scaffold6 | 679 |
| Gpal_D383_g06840 | SP | SMD<SMI | 1.76 | NA | scaffold7 | 717 |
| Gpal_D383_g06902 | SP | SMD<SMI | 1.64 | NA | scaffold7 | 652 |
| Gpal_D383_g07012 | NA | SMD<SMI | 1.37 | EGT36195.1CBN-BED-2 protein | scaffold7 | 3221 |
| Gpal_D383_g07968 | NA | SMD>SMI | 3.95 | NA | scaffold8 | 279 |
| Gpal_D383_g07991 | SP | SMD<SMI | 1.67 | AEL16453.1VAP1 protein | scaffold8 | 1124 |
| Gpal_D383_g08056 | SP | SMD<SMI | 1.7 | NA | scaffold8 | 1041 |
| Gpal_D383_g08123 | NA | SMD<SMI | 1.43 | CAD2180188.1unnamed protein product | scaffold9 | 1680 |
| Gpal_D383_g08222 | NA | SMD>SMI | 1.44 | NA | scaffold9 | 845 |
| Gpal_D383_g08426 | SP | SMD>SMI | 3.09 | NA | scaffold9 | 1259 |
| Gpal_D383_g08460 | NA | SMD<SMI | 1.5 | XP_024502236.1cAMP-dependent protein kinase catalytic subunit PRKX | scaffold9 | 2194 |
| Gpal_D383_g08466 | SP | SMD<SMI | 1.82 | NA | scaffold9 | 1375 |
| Gpal_D383_g08467 | NA | SMD<SMI | 1.93 | NA | scaffold9 | 2076 |
| Gpal_D383_g08470 | NA | SMD<SMI | 1.95 | NA | scaffold9 | 4678 |
| Gpal_D383_g08475 | SP | SMD<SMI | 2.08 | NA | scaffold9 | 738 |
| Gpal_D383_g08484 | SP | SMD<SMI | 1.6 | NA | scaffold9 | 1375 |
| Gpal_D383_g08485 | SP | SMD<SMI | 2.65 | NA | scaffold9 | 321 |
| Gpal_D383_g08486 | NA | SMD<SMI | 1.69 | NA | scaffold9 | 3093 |
| Gpal_D383_g08490 | SP | SMD<SMI | 1.97 | NA | scaffold9 | 735 |
| Gpal_D383_g08491 | SP | SMD<SMI | 1.84 | NA | scaffold9 | 225 |
| Gpal_D383_g08500 | NA | SMD>SMI | 22.8 | NA | scaffold9 | 117 |
| Gpal_D383_g08501 | SP | SMD>SMI | 31,00 | NA | scaffold9 | 3485 |
| Gpal_D383_g08568 | SP | SMD>SMI | 2.98 | AAN15808.1putative esophageal gland cell secretory protein 28 | scaffold9 | 2384 |
| Gpal_D383_g09150 | SP | SMD>SMI | 2.72 | NA | scaffold9 | 658 |
| Gpal_D383_g09174 | NA | SMD>SMI | 2.94 | NA | scaffold9 | 2122 |
| Gpal_D383_g09177 | SP | SMD>SMI | 1.69 | AVA09710.1putative effector protein | scaffold9 | 970 |
| Gpal_D383_g09807 | SP | SMD<SMI | 1.59 | NA | scaffold10 | 651 |
| Gpal_D383_g09921 | SP | SMD<SMI | 2.21 | AAC48326.1beta-1,4-endoglucanase-2 precursor | scaffold10 | 1458 |
| Gpal_D383_g09988 | NA | SMD>SMI | 2.02 | NA | scaffold10 | 2228 |
| Gpal_D383_g10530 | NA | SMD<SMI | 10.4 | NA | scaffold12 | 1482 |
| Gpal_D383_g10744 | NA | SMD>SMI | 413.00 | CAD2191927.1unnamed protein product | scaffold12 | 2251 |
| Gpal_D383_g10745 | NA | SMD>SMI | 8.56 | NA | scaffold12 | 2747 |
| Gpal_D383_g10764 | NA | SMD<SMI | 2.53 | NA | scaffold12 | 3938 |
| Gpal_D383_g10769 | NA | SMD>SMI | 3.34 | NA | scaffold12 | 1967 |
| Gpal_D383_g10792 | NA | SMD>SMI | 58.00 | NA | scaffold12 | 883 |
| Gpal_D383_g10795 | NA | SMD>SMI | 2.66 | NA | scaffold12 | 6558 |
| Gpal_D383_g10798 | NA | SMD>SMI | 2.09 | NA | scaffold12 | 2640 |
| Gpal_D383_g10941 | SP | SMD>SMI | 4.23 | NA | scaffold13 | 3427 |
| Gpal_D383_g11042 | SP | SMD<SMI | 1.81 | AGI97007.1ubiquitin extension protein UBI-2 | scaffold13 | 345 |
| Gpal_D383_g11079 | NA | SMD>SMI | 5.62 | CAP39245.2Protein CBG22717 | scaffold13 | 612 |
| Gpal_D383_g11128 | SP | SMD<SMI | 2.47 | KHN77175.1Putative carbonic anhydrase 5 | scaffold13 | 2248 |
| Gpal_D383_g11164 | SP | SMD<SMI | 1.72 | CAD2122873.1unnamed protein product | scaffold13 | 1423 |
| Gpal_D383_g11358 | SP | SMD<SMI | 1.79 | AAM93256.1heat shock protein 70-C | scaffold13 | 1980 |
| Gpal_D383_g11401 | SP | SMD<SMI | 1.93 | NA | scaffold13 | 1957 |
| Gpal_D383_g11619 | NA | SMD>SMI | 2.5 | XP_029721750.1protein lethal(2)essential for life-like | scaffold14 | 781 |
| Gpal_D383_g11910 | SP | SMD<SMI | 1.94 | RWD38789.1glycosyltransferase family 4 protein | scaffold15 | 2139 |
| Gpal_D383_g11913 | NA | SMD>SMI | 3.19 | NA | scaffold15 | 891 |
| Gpal_D383_g12128 | SP | SMD<SMI | 1.96 | WP_067658468.1nucleoside hydrolase | scaffold15 | 1727 |
| Gpal_D383_g12331 | SP | SMD<SMI | 2.03 | NA | scaffold16 | 1363 |
| Gpal_D383_g12349 | NA | SMD<SMI | 1.84 | AVA09730.1putative effector protein | scaffold16 | 2412 |
| Gpal_D383_g12448 | SP | SMD<SMI | 2.82 | ACO35734.1RBP-5 protein | scaffold16 | 765 |
| Gpal_D383_g12491 | SP | SMD>SMI | 2.1 | CDJ96194.1Protein LRON10 | scaffold16 | 1186 |
| Gpal_D383_g13464 | SP | SMD<SMI | 1.77 | NA | scaffold19 | 1288 |
| Gpal_D383_g13465 | SP | SMD<SMI | 1.86 | NA | scaffold19 | 3006 |
| Gpal_D383_g13538 | NA | SMD<SMI | 3.47 | NA | scaffold19 | 1581 |
| Gpal_D383_g13546 | NA | SMD>SMI | 2.77 | XP_029200799.1dual specificity mitogen-activated protein kinase kinase 5-like | scaffold19 | 3969 |
| Gpal_D383_g13636 | NA | SMD<SMI | 2.46 | KAF7640168.1LIM zinc-binding domain-containing protein | scaffold20 | 623 |
| Gpal_D383_g13665 | NA | SMD>SMI | 1.97 | NA | scaffold20 | 1704 |
| Gpal_D383_g13713 | NA | SMD<SMI | 2.37 | NA | scaffold20 | 4996 |
| Gpal_D383_g14389 | NA | SMD<SMI | 1.8 | KAF0305826.1LIM/homeobox protein Lhx3 | scaffold26 | 2364 |
| Gpal_D383_g14606 | SP | SMD<SMI | 1.78 | NA | scaffold27 | 968 |
| Gpal_D383_g14646 | NA | SMD<SMI | 4.22 | CAD2202613.1unnamed protein product | scaffold28 | 6435 |
| Gpal_D383_g15097 | NA | SMD<SMI | 2.03 | NA | scaffold34 | 438 |
| Gpal_D383_g15176 | SP | SMD<SMI | 2.22 | AEA08843.1pectate lyase 1 | scaffold34 | 795 |
| Gpal_D383_g15260 | NA | SMD>SMI | 6.48 | AVA09700.1putative effector protein | scaffold35 | 815 |
| Gpal_D383_g15296 | NA | SMD<SMI | 2.8 | NA | scaffold35 | 5365 |
| Gpal_D383_g15313 | SP | SMD<SMI | 2.33 | AFN86179.1secreted SPRY domain-containing protein 18 | scaffold35 | 1465 |
| Gpal_D383_g15316 | SP | SMD<SMI | 2.57 | AFN86180.1secreted SPRY domain-containing protein 19 | scaffold35 | 1502 |
| Gpal_D383_g15527 | SP | SMD<SMI | 1.7 | NA | scaffold38 | 468 |
| Gpal_D383_g15697 | NA | SMD<SMI | 1.48 | NA | scaffold40 | 3627 |
| Gpal_D383_g16157 | NA | SMD>SMI | 1.4 | OZC12308.1small nuclear ribonucleoprotein Sm D3 family protein | scaffold44 | 667 |
| Gpal_D383_g16202 | NA | SMD<SMI | 2.02 | NA | scaffold44 | 676 |
| Gpal_D383_g16477 | SP | SMD<SMI | 2.41 | AFN86174.1secreted SPRY domaincontaining protein 5 | scaffold50 | 1979 |
| Gpal_D383_g16577 | NA | SMD>SMI | 1.47 | NA | scaffold51 | 2292 |
| Gpal_D383_g16861 | NA | SMD<SMI | 1.87 | NA | scaffold54 | 480 |
| Gpal_D383_g16918 | SP | SMD<SMI | 1.74 | AHW98762.1beta-1,4-endoglucanase precursor | scaffold54 | 1272 |
| Gpal_D383_g17133 | SP | SMD<SMI | 2.2 | ACU64858.1pectate lyase 2 | scaffold57 | 756 |
| Gpal_D383_g17812 | SP | SMD<SMI | 2.03 | OQV16814.1Phosphatidylinositol 5-phosphate 4-kinase type-2 alpha | scaffold91 | 3654 |

**Fig. S1 –** 2-dimensional reduction separating SMD lineages from SMI lineages with (A) or without (B) samples SMD2A and SMI3A.

A


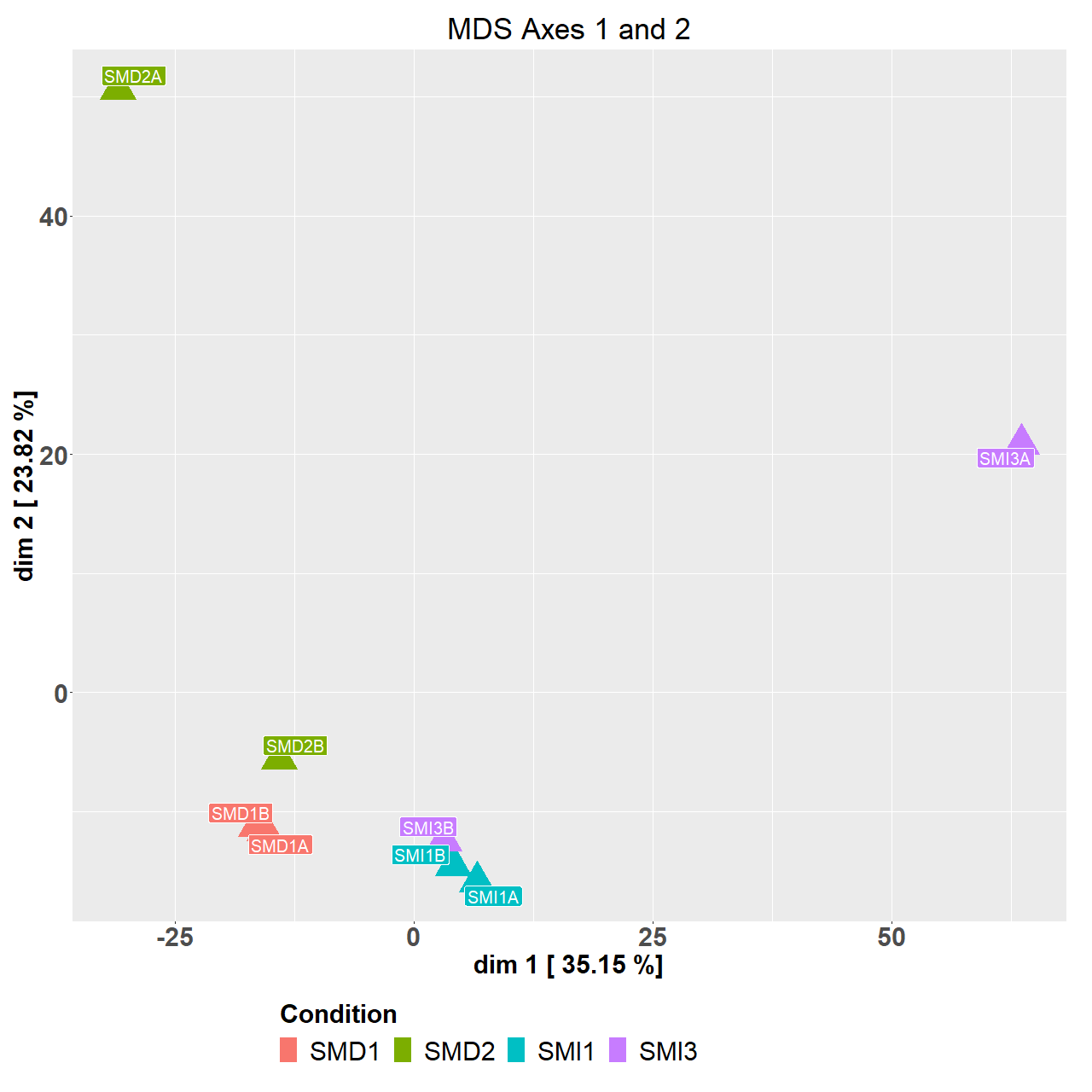


B


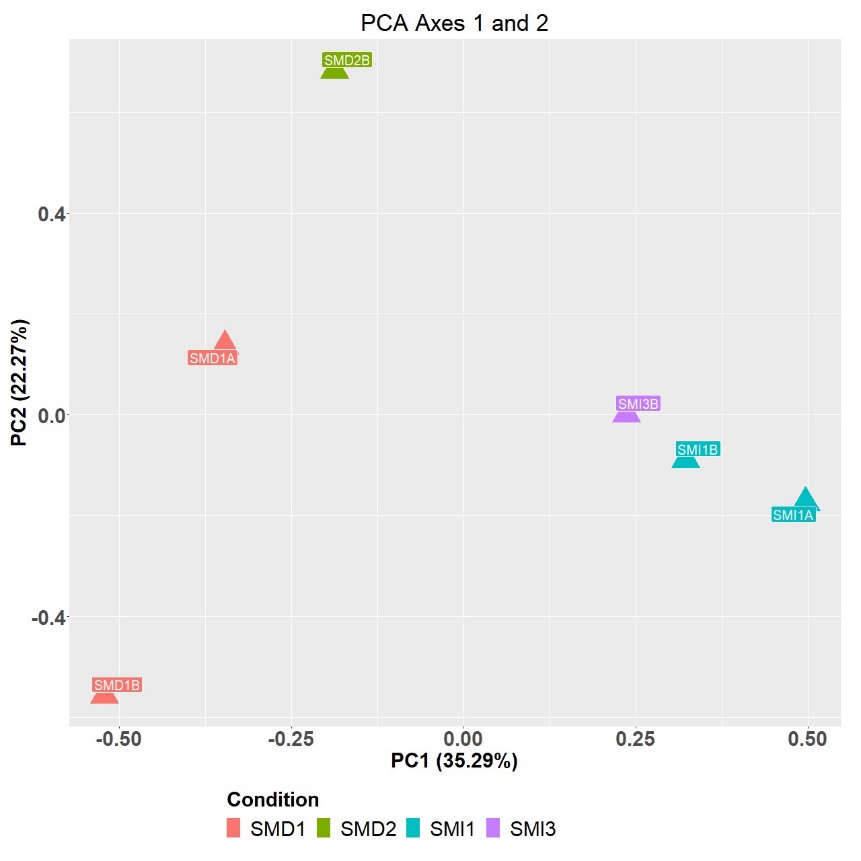


**Fig. S2 –** Heatmap clustering of samples based on CPM counts per sample.


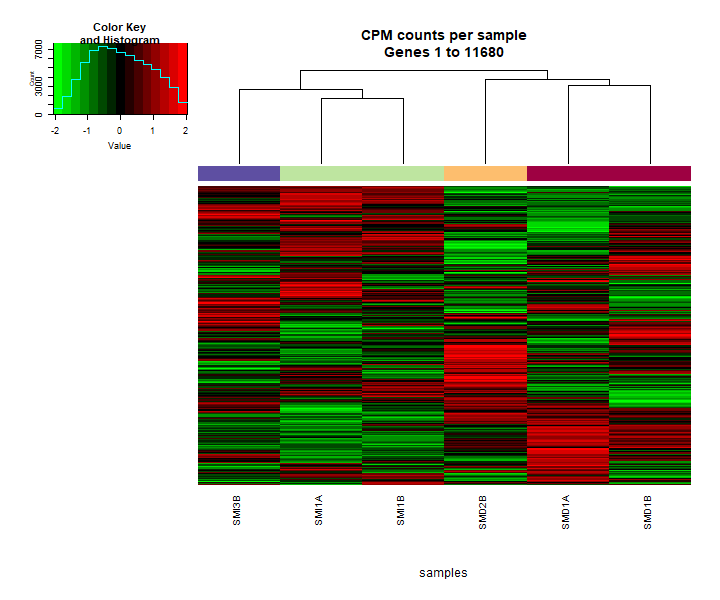


**Fig. S3 –** GO-term enrichment on the 69 DEGs up-regulated in SMI lineages. Y-axis represents enriched GO-terms for each ontology category (MF = Molecular Function, CC = Cellular Component and BP = Biological Process). X-axis shows p-values for each enriched GO-term. A larger circle diameter highlights higher number of genes that have enriched the term.


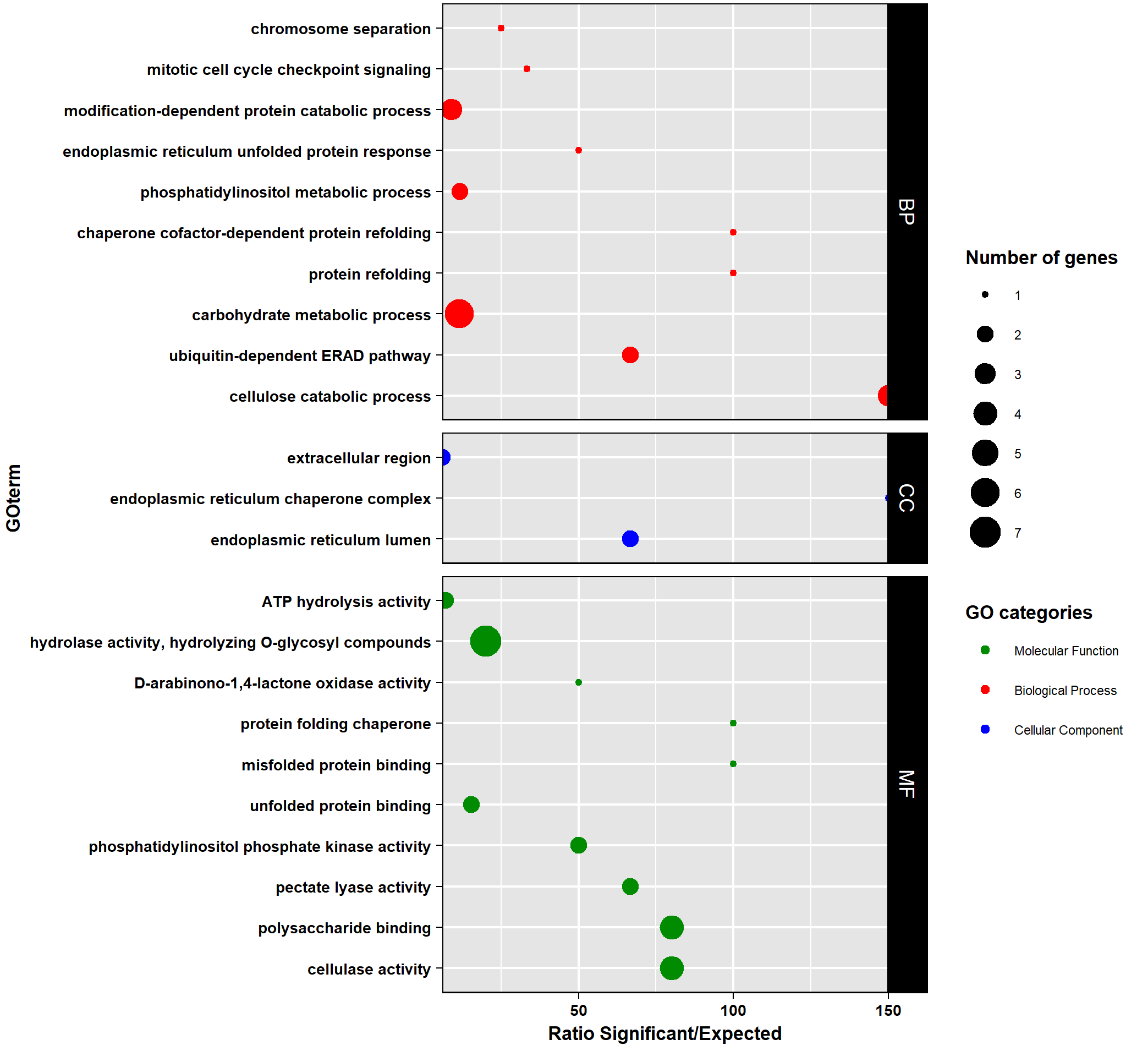


**Fig. S4 –** Protocol for phenotyping the virulence level of different lineages.


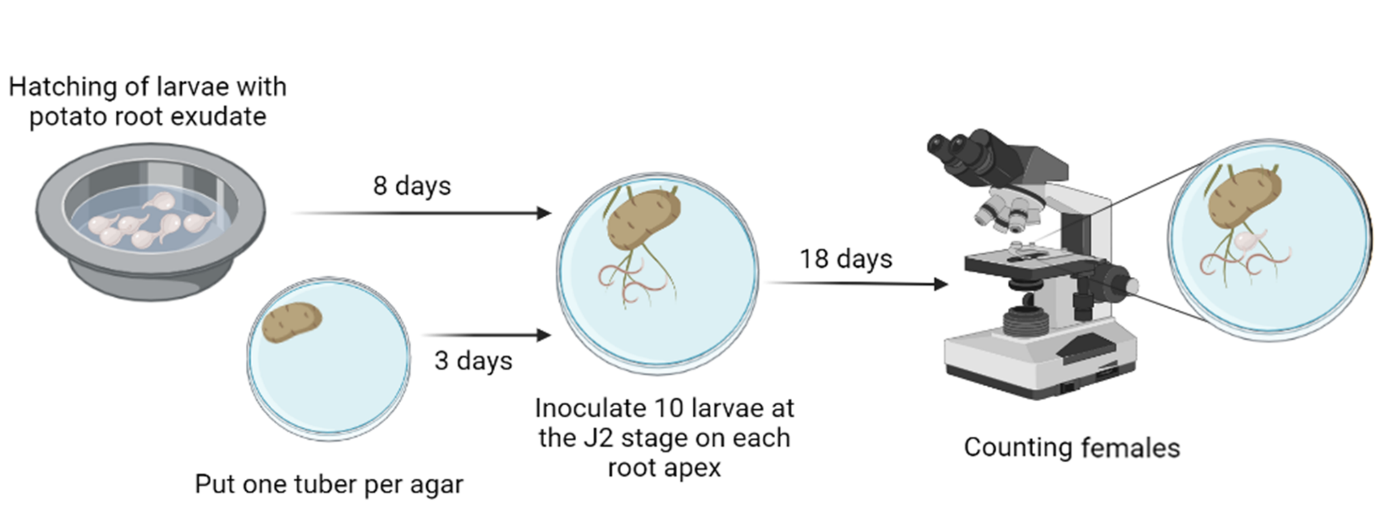

Supplement: Supplementary file 1 — Supplementary Material 1. [file 12864_2025_11332_MOESM1_ESM.docx]
